# Magnesium-Based Membrane for Alveolar Ridge Regeneration—A Scoping Review

**DOI:** 10.3390/jfb17060293

**Published:** 2026-06-12

**Authors:** Dragana Gabrić, Yuval Reiser, Ivica Pelivan, Igor Smojver, Luka Marković

**Affiliations:** 1 Department of Oral Surgery, School of Dental Medicine, University of Zagreb, HR-10000 Zagreb, Croatia; dgabric@sfzg.unizg.hr (D.G.); ismojver@gmail.com (I.S.); 2 Clinical Hospital Center Zagreb, University Dental Clinic, HR-10000 Zagreb, Croatia; 3 School of Dental Medicine, University of Zagreb, HR-10000 Zagreb, Croatia; yreiser@sfzg.unizg.hr (Y.R.); markovi.luka@gmail.com (L.M.); 4 Department of Removable Prosthodontics, School of Dental Medicine, University of Zagreb, HR-10000 Zagreb, Croatia

**Keywords:** biomaterial, bone regeneration, guided bone regeneration, magnesium, regenerative dentistry, resorbable metal

## Abstract

Magnesium-based membranes are promising biomaterials for guided bone regeneration due to their unique properties of mechanical strength, biocompatibility, and controlled biodegradation. This scoping review aimed to map and synthesize the available evidence regarding the use of magnesium-based membranes and fixation screws in alveolar ridge regeneration and guided bone regeneration procedures. Relevant studies were identified through a literature search conducted from November 2025 to May 2026, using several databases following the Preferred Reporting Items for Systematic reviews and Meta-Analyses extension for Scoping Reviews guidelines. Thirty-nine studies met the inclusion criteria, including in vitro studies, preclinical animal studies, clinical case reports and case series, and narrative or systematic reviews. In vitro studies demonstrated cytocompatibility and fibroblast adhesion, while moderate magnesium ion concentrations increased markers of osteogenic differentiation. Preclinical animal studies reported controlled degradation, biocompatible tissue responses, maintenance of barrier function during early healing, and findings suggesting potential osteogenic stimulation. Clinical evidence, limited to case reports and small case series, described the use of magnesium membranes in horizontal and vertical ridge augmentation, sinus lift procedures, immediate dentoalveolar regeneration, periodontal defects, and cystic lesions, with generally uneventful healing outcomes and preserved bone volume. Reported complications were mainly minor and included transient soft tissue reactions, membrane exposure, and localized gas cavity formation. However, the available evidence remains limited to low-level studies without controlled clinical trials. Current findings are insufficient to establish clinical efficacy or superiority over conventional membranes, highlighting the need for larger prospective controlled studies. The review’s findings could help researchers advance the understanding of bone regeneration and help develop new strategies to improve and further investigate bone regeneration.

## 1. Introduction

An alveolar bone defect is defined as a localized loss of the bone supporting the teeth and forming the dental arch, presenting as horizontal, vertical, or circumferential defects [1]. Bone resorption often arises from multiple causes, including the most frequent factors: tooth extraction [1,2], periodontal inflammation [3], tumors, cysts [4,5], and trauma [5]. To mitigate bone loss, reconstructive approaches, broadly referred to as alveolar ridge regeneration, such as ridge expansion, distraction osteogenesis, sandwich osteotomy, and guided bone regeneration (GBR), are essential for modern implant and prosthetic rehabilitation [6]. Among these, GBR is the most widely documented and clinically utilized approach. GBR is a specific surgical technique based on the biological principle of cellular exclusion: a biocompatible barrier membrane is placed over the bone defect to physically prevent the ingrowth of faster-proliferating epithelial and connective tissue cells, thereby creating a protected space in which osteogenic cells can migrate, proliferate, and form new bone [7,8]. Ultimately, the success of this process depends on achieving tight contact between the graft and host bone, mechanical stability, and an adequate blood supply to support predictable bone augmentation [9].

Successful bone regeneration relies on coordinated cellular and molecular events. Healing begins with an inflammatory phase in which macrophages and immune cells regulate the recruitment and osteogenic differentiation of mesenchymal stem cells (MSCs) derived primarily from the periosteum and bone marrow 8]. This process is governed by key signaling pathways, including Wnt/β-catenin and BMP/SMAD, with macrophage-MSC crosstalk mediated by paracrine factors such as BMP-2 playing a central role in osteogenesis [10]. Biomaterials support these processes by providing osteoconductive scaffolds for cell attachment and migration, delivering osteoinductive cues, maintaining space to prevent soft-tissue invasion, and modulating the local osteoimmune microenvironment [11]. In guided bone regeneration, barrier membranes act as bioactive compartments that concentrate growth factors (BMP-2, FGF-2, and VEGF) and guide cellular events within the defect, while bone graft materials must exhibit biocompatibility, appropriate porosity, mechanical integrity, and controlled biodegradation to achieve predictable bone formation [11].

The membrane used for GBR is an essential component of the treatment and is typically classified as either first-generation non-resorbable materials, such as expanded polytetrafluoroethylene (e-PTFE), or second-generation resorbable membranes, most commonly collagen [9,12]. While non-resorbable membranes provide good biocompatibility and stability, thereby maintaining good space maintenance, they require a second surgery for removal. Resorbable membranes were therefore introduced to eliminate the need for membrane retrieval and have since become widely used across many clinical applications [12]. Collagen membranes, while highly biocompatible and resorbable, exhibit limited mechanical strength and unpredictable degradation rate, which can compromise space maintenance and barrier function [13].

Given the limitations of resorbable membranes in mechanical strength and predictable degradation, magnesium has been investigated as a potential alternative material [14]. Magnesium is a naturally occurring element essential for bone metabolism, and its biodegradability, mechanical strength, and potential bioactivity make it an attractive candidate for resorbable membrane applications [14]. The scope of this review encompasses barrier membranes in which magnesium constitutes the primary structural component, including pure magnesium membranes, magnesium alloy membranes, and magnesium-reinforced composite membranes (e.g., magnesium–polymer constructs) designed for use in GBR. Conventional membranes that are merely doped or surface-functionalized with magnesium ions without magnesium serving as the primary structural material are outside the scope of this review.

The translational development of magnesium membranes has followed the standard biomaterial evaluation pathway. In vitro studies first established cytocompatibility, cell adhesion, and osteogenic differentiation potential on magnesium-based surfaces, suggesting that magnesium degradation products may enhance osteogenic signaling at appropriate concentrations [15]. Subsequently, preclinical in vivo studies in animal models evaluated barrier function, degradation behavior, tissue response, and bone formation, suggesting that magnesium membranes may maintain space during the critical early healing phase and undergo gradual resorption [16,17]. Clinical evidence has thus far been limited to case reports and small case series documenting the use of magnesium membranes across various indications; however, a systematic synthesis of existing evidence is lacking, and all available evidence consists of Level 4–5 studies (case reports, preclinical investigations, and expert reviews) without controlled clinical trials.

Critical knowledge gaps include the optimal degradation kinetics, comparative efficacy versus conventional membranes (which have never been directly compared in RCTs), management of complications such as gas cavity formation and membrane exposure, and long-term clinical outcomes. Therefore, this scoping review aims to systematically map and critically appraise the existing literature on magnesium-based barrier membranes for guided bone regeneration, with explicit attention to evidence quality and the distinction between preliminary findings and established clinical practice. The review identifies current evidence gaps and highlights the need for larger, prospective, controlled clinical trials to determine whether magnesium membranes provide clinically meaningful advantages over conventional materials. The key finding of this review is that while preliminary evidence supports the biological plausibility and regenerative potential of magnesium membranes, the current body of evidence remains insufficient to establish clinical efficacy, recommend magnesium over conventional materials, or define standardized clinical protocols.

## 2. Materials and Methods

### 2.1. Protocol Development and Registration

This scoping review adhered to the Preferred Reporting Items for Systematic reviews and Meta-Analyses extension for Scoping Reviews (PRISMA-ScR) guidelines [18]. The completed PRISMA-ScR checklist is provided as Supplementary Material File Table S1. The protocol was prospectively registered with the Open Science Framework (OSF) public registry (Registration DOI: doi.org/10.17605/OSF.IO/CG9JK).

### 2.2. Eligibility Criteria

Eligibility criteria were developed using the Population–Concept–Context (PCC) framework, as recommended for scoping reviews by the Joanna Briggs Institute (JBI):

- Population: Patients, animal models, or cell cultures relevant to alveolar ridge defects or alveolar bone regeneration.
- Concept: Use of magnesium-based barrier membranes (pure magnesium, magnesium alloy, or magnesium-reinforced composite membranes), with or without magnesium fixation screws, for guided bone regeneration.
- Context: Oral and maxillofacial surgery, implant dentistry, periodontics, and guided bone regeneration procedures involving the alveolar ridge.

#### 2.2.1. Inclusion Criteria

Studies were eligible for inclusion if they met the following criteria:

- Topic: Investigated magnesium-based barrier membranes (pure magnesium, magnesium alloy, or magnesium-reinforced composite membranes), with or without magnesium fixation screws, for alveolar bone regeneration or guided bone regeneration.
- Study design: In vitro studies, preclinical in vivo (animal) studies, clinical studies, and review articles addressing magnesium membranes in alveolar bone regeneration.
- Outcomes: Reported at least one outcome relevant to bone regeneration, membrane degradation, biocompatibility, mechanical properties, or clinical performance.
- Language: Publications written in English.

#### 2.2.2. Exclusion Criteria

Studies were excluded based on the following criteria:

- Off-topic studies: Studies not involving magnesium-based membranes.
- Non-relevant anatomical context: Studies focused solely on orthopedic, cardiovascular, or non-alveolar bone applications.
- Non-relevant outcomes: Studies lacking relevant outcome measures for bone regeneration or clinical application.

Orthopedic bone healing differs from alveolar bone with respect to embryological origin, biomechanical loading, vascularization, and healing patterns [19]; therefore, findings from orthopedic applications were considered not directly translatable to guided bone regeneration or implant dentistry and were excluded.

### 2.3. Search Strategy

A comprehensive literature search was conducted between 29 November 2025 and 16 January 2026 using the following electronic databases: PubMed, Google Scholar, Scopus, Cochrane Library, Web of Science, and ScienceDirect. The search strategy was guided by the PCC framework and aimed to identify studies addressing alveolar bone and tissue regeneration associated with magnesium-based biomaterials.

The following search string was applied across all databases:

(“Bone Regeneration”[Title/Abstract] OR “Alveolar Bone Grafting”[Title/Abstract] OR “Tissue Regeneration”[Title/Abstract]) AND “Magnesium”[Title/Abstract].

The complete database-specific search strategy is summarized in Table 1.

Eligible studies were identified according to predefined inclusion and exclusion criteria. After removal of duplicate records, titles and abstracts were independently screened by two reviewers. Full-text articles of potentially eligible studies were subsequently assessed independently by the same reviewers to determine final inclusion.

Following peer review, a supplementary search was performed on 3–5 May 2026 to address a potential gap in the original strategy. The updated search incorporated additional terms central to guided bone regeneration: (“Bone Regeneration” OR “Alveolar Bone Grafting” OR “Tissue Regeneration” OR “Guided Bone Regeneration” OR “GBR” OR “Ridge Augmentation” OR “Alveolar Ridge Augmentation”) AND (“Magnesium” OR “Mg” OR “Magnesium alloy” OR “Magnesium-based”) AND (“Membrane” OR “Barrier Membrane” OR “Resorbable Membrane” OR “Biodegradable Membrane” OR “Barrier” OR “Mesh”).

### 2.4. Quality Assessment

The quality of evidence was assessed using the Oxford Center for Evidence-Based Medicine (CEBM) levels of evidence [20], as follows:

- Level 1: Systematic reviews, meta-analyses, randomized controlled trials.
- Level 2: Cohort studies, low-quality randomized controlled trials.
- Level 3: Case–control studies.
- Level 4: Case series, case reports, and poor-quality cohort/case–control studies.
- Level 5: Expert opinion, mechanism-based reasoning, and clinical guidelines.

### 2.5. Data Extraction

Relevant data were individually extracted from the selected articles by two reviewers using separate customized charts. Information such as authors’ names, title, year of publication, study type, sample size, and important findings was categorized and summarized. All studies were published between 2013 and 2026.

## 3. Results

### 3.1. Study Selection

**Figure 1:**
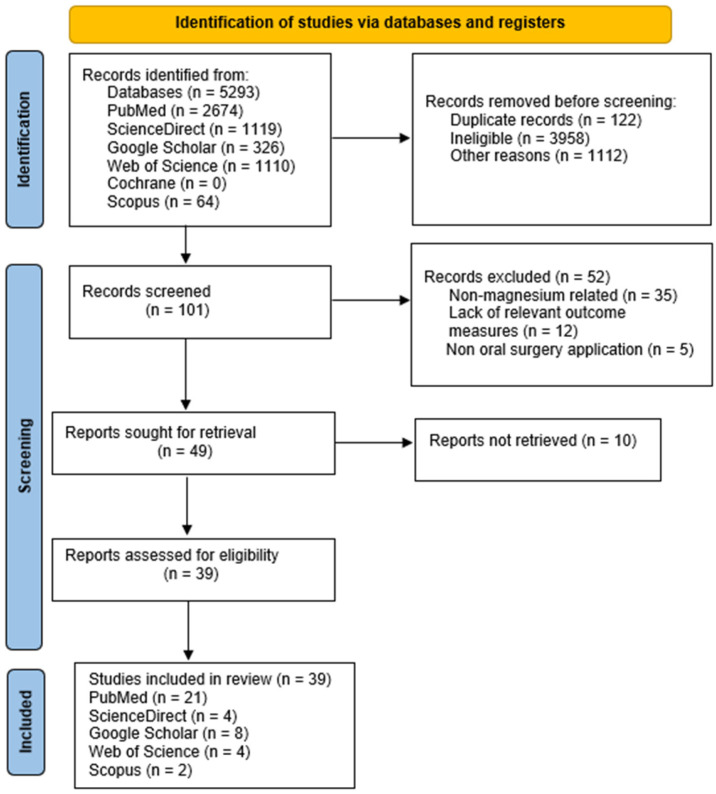
Preferred Reporting Items for Systematic reviews and Meta-Analyses flowchart illustrating the identification, screening, and selection process of the records during the search.

The PRISMA 2020 flow diagram illustrates the study selection process (Figure 1).

Searches in PubMed, Scopus, Web of Science, and ScienceDirect were exported and screened using Rayyan AI (Rayyan 1.4.3, Rayyan Systems Inc., Cambridge, MA, USA), automation-assisted deduplication, and blinded dual-review screening. Due to Google Scholar’s proprietary ranking algorithm and lack of reproducible search functionality, only the first 200 results were screened, as this threshold has been shown to capture the majority of relevant citations while maintaining feasibility [21]. Results were screened in the order presented by Google Scholar on the search date. Google Scholar was queried using the Advanced Search interface, applying the “exact words” option within the search engine settings (exact phrase matching enabled). The screening process resulted in 70 records assessed at the title and abstract level. Following an independent review, 30 records were excluded (24 non-magnesium-membrane-related, 3 lacking relevant outcome measures, and 3 involving orthopedic applications). Thus, 40 full-text reports were sought for retrieval, but 10 were not retrieved, resulting in 30 studies assessed for eligibility. After full-text, 30 studies (PubMed, n = 15; ScienceDirect, n = 3; Google Scholar, n = 7; Web of Science, n = 3; Scopus, n = 2) published between 2013 and 2025 met the inclusion criteria and were included in the final review.

The supplementary search identified 2243 additional records that were exported and screened using Rayyan AI, of which 24 were duplicates of studies already included in the original search, 2210 were new records that did not meet inclusion criteria upon screening, and 9 new studies met inclusion criteria and were added to the review (PubMed, n = 6; ScienceDirect, n = 1; Google Scholar, n = 1; Web of Science, n = 1).

Study selection was conducted independently by two reviewers (I.P. and Y.R.). Disagreements during title/abstract screening and full-text assessment were resolved through discussion until consensus was reached. In cases where consensus could not be achieved through discussion alone, a third reviewer (D.G.) was consulted for final adjudication. Inter-rater agreement was not formally calculated.

When systematic or narrative reviews were identified during screening, they were included for descriptive purposes, and their reference lists were systematically screened to identify additional primary studies meeting the inclusion criteria. This process identified four primary studies of interest; however, all were subsequently excluded due to duplication.

### 3.2. Data Synthesis

Given the heterogeneity of the included studies, a qualitative narrative synthesis was used. To provide clarity and structure, the findings were grouped according to study types: preclinical animal studies, in vitro investigations, case reports, and case series.

### 3.3. Evidence Quality Distribution (Oxford CEBM)

The evidence was classified as Levels 1, 2, 3, 4, and 5 in 0 (0%), 0 (0%), 0 (0%), 13 (33%), and 26 (67%) studies, respectively. It should be noted that the Oxford CEBM hierarchy was designed for clinical research questions, and its application to preclinical studies presents an inherent limitation. Under this framework, both expert opinion and controlled preclinical experiments receive the same Level 5 classification, despite representing epistemologically distinct evidence types. This classification was applied descriptively to map the evidence landscape rather than to imply equivalence between these study types.

This predominance of low-level evidence (33% Level 4 case series, 67% Level 5 expert opinions, and preclinical and in vitro studies) is a critical limitation that directly impacts the strength of conclusions that can be drawn from this review. Case reports and case series (Level 4) document clinical outcomes but lack control groups, randomization, and standardized outcome measures, limiting the ability to assess true efficacy or compare magnesium membranes to conventional alternatives. Preclinical and in vitro studies (Level 5), while mechanistically informative, cannot directly translate to clinical efficacy in humans. The absence of Level 1–3 evidence (randomized trials, cohort studies, systematic reviews) indicates that no controlled clinical comparison data exist, and current clinical use is based on observational case experience and extrapolation from preclinical models. Consequently, claims regarding “reliability,” “predictability,” or clinical superiority cannot be supported by the current evidence base.

As this study was designed as a scoping review, the aim was to map existing evidence rather than assess intervention effects; therefore, a formal risk-of-bias assessment was not applied, in line with JBI and PRISMA-ScR guidance, where bias scoring is not mandatory for evidence-mapping reviews. Since most available magnesium membrane studies in oral bone regeneration are Level 4–5 evidence (case reports, small series, preclinical/in vitro), their methodological limitations, namely small sample sizes, lack of controlled trials, heterogeneity, and limited generalizability, will instead be reported transparently in Section 4 rather than used as exclusion criteria.

### 3.4. Study Characteristics

The 39 studies included one systematic review, six narrative reviews, one literature review and case series, four in vitro studies, nine original in vivo studies, six preclinical in vivo and in vitro research articles, one retrospective case series, six clinical case reports, and five case series (Table 2).

#### 3.4.1. In Vitro Studies

In vitro studies evaluated cell adhesion, migration, viability, and osteogenic differentiation on magnesium-based surfaces or in the presence of magnesium ions [15,22]. Human gingival fibroblasts demonstrated attachment and migration on biodegradable magnesium substrates [15]. Osteogenic assays reported increased alkaline phosphatase activity, upregulation of osteogenic gene expression, and higher mineralization-related activity at moderate magnesium ion concentrations, while higher concentrations were associated with reduced cell viability [22,24]. Composite magnesium-reinforced polylactide membranes demonstrated significantly higher maximum load and stiffness compared to non-reinforced polylactide, with the magnesium alloy core undergoing complete degradation between 16 and 20 weeks without adversely affecting pre-osteoblast cell viability [22]. In the broader magnesium biomaterial field, walnut shell-filled polylactic acid–hydroxyapatite hybrid coatings applied to magnesium substrates demonstrated improved adhesion strength, significantly reduced corrosion current density, and enhanced in vitro bioactivity through promotion of calcium- and phosphorus-rich layer formation on the coating surface [23]. Additionally, a novel alginate–CuO–MXene (Ti3C2Tx) hybrid coating applied to AZ31 magnesium alloy achieved a 77% reduction in corrosion current density compared to uncoated magnesium while demonstrating broad-spectrum antibacterial activity and maintaining osteoblastic cell viability and favorable cell adhesion [24].

#### 3.4.2. Preclinical In Vivo Studies

Preclinical in vivo studies investigated magnesium-based membranes and fixation devices primarily in animal models, including beagle dogs and rabbits [15,16,17,25,26,27,28,29,30,31,32,33]. The evaluated outcomes included membrane degradation behavior, tissue response, space maintenance, improved bone–implant contact, favorable tissue responses, and new bone formation assessed by histology and micro-computed tomography [16,28,29,34]. Magnesium membranes maintained barrier function during the early healing phase and underwent gradual degradation, with near-complete resorption reported between 16 and 52 weeks, depending on membrane composition and surface treatment [26,27,35]. Bone formation beneath magnesium membranes was comparable to that observed under collagen or titanium barriers in all reported models [16,28,31]. Early healing, however, may be slower with a heightened immune response, suggesting a trade-off between osteogenesis and soft tissue recovery [36]. More recent preclinical studies have expanded magnesium-based membranes toward composite and architecturally optimized designs, including 3D-printed magnesium ammonium phosphate/polycaprolactone and magnesium-reinforced sandwich-structured membranes, which demonstrated controlled degradation, mechanical stability, and enhanced osteogenic performance in preclinical models [36,37]. A magnesium-reinforced collagen membrane with metal-phenolic network-coated micro-magnesium wires demonstrated sustained release of molecular hydrogen and magnesium ions, attenuating intracellular reactive oxygen species and reprogramming cellular metabolism from glycolysis toward oxidative phosphorylation via pathway activation, with in vivo rat calvarial defect models confirming significantly promoted osteogenesis [38]. Evaluation of biodegradable magnesium metal GBR membranes combined with bovine graft, with and without hyaluronate, reported favorable bone regeneration outcomes, suggesting potential synergistic effects between magnesium membranes and adjunctive biomaterials [39].

#### 3.4.3. Clinical Case Reports and Case Series

Clinical evidence consisted of case reports and small case series describing the use of magnesium membranes in horizontal and vertical ridge augmentation, immediate dentoalveolar regeneration, sinus lift procedures, cystic lesions, and intrabony periodontal defects [40,41,42,43,44,45,46,47,48,49,50,51]. Reported outcomes included radiographic bone gain, preservation of graft volume at re-entry, successful implant placement, and complete membrane resorption [41,42,43,44,45,46,47,48]. Follow-up durations ranged from 3 months to 2 years [42,43].

#### 3.4.4. Reported Complications

Reported complications were limited and mainly included transient soft-tissue reactions, membrane exposure, and localized gas cavity formation during membrane degradation [28,41,43,48,50]. No infections or graft failures were reported across the included clinical studies [41,42,43,44,45,46,47,48,49,50]. In cases of membrane exposure, regenerated bone volume was preserved, and implant placement proceeded as planned [48,49].

## 4. Discussion

This scoping review synthesized evidence from 39 studies evaluating magnesium-based membranes and fixation devices for alveolar bone regeneration. The available in vitro investigations and preclinical in vivo studies in animal models (Level 5, 67% of included studies) demonstrated cellular responses, membrane degradation characteristics, and bone formation under controlled laboratory conditions. Nevertheless, these findings cannot be directly translated to clinical efficacy in humans without prospective clinical investigation. Clinical evidence, consisting exclusively of case reports and small case series (Level 4, 33% of included studies), documents clinical outcomes including bone regeneration across various indications (ridge augmentation, sinus procedures, cystic defects, and periodontal lesions). However, without control groups, randomization, or standardized outcome measures, these reports provide observational experience rather than evidence of efficacy or safety. Reported complications in case series were generally minor. However, the limited sample sizes and lack of systematic adverse event monitoring prevent a comprehensive safety assessment. Overall, the current evidence is insufficient to establish clinical efficacy, superiority over conventional materials, or standardized clinical protocols. The predominance of Level 4–5 evidence, absence of Level 1–3 studies (RCTs, cohort studies, systematic reviews with meta-analysis), and small sample sizes in all clinical reports indicate that magnesium membranes remain at an early investigative stage, and conclusions regarding clinical utility must be considered preliminary pending larger, controlled clinical trials.

Bone regeneration is a complex challenge, both physiologically and technically [43]. For implant placement, clinicians may opt for short implants or a staged two-step procedure. However, achieving optimal implant positioning often requires prior bone augmentation. GBR is commonly used to increase vertical and horizontal bone volume, typically employing titanium-reinforced membranes or meshes for mechanical support [7,12]. While resorbable collagen membranes are an option, they may collapse during vertical augmentation without reinforcement, thereby reducing the final bone gain, underscoring the need for strategies that provide both stability and biological support [43]. To address the limitations of conventional resorbable membranes in GBR, a novel resorbable magnesium membrane (NOVAMag^®^ membrane, Botiss biomaterials GmbH, Berlin, Germany) has been developed [16].

### 4.1. Biological Activity and Effect of Magnesium Ion Release

Beyond mechanical considerations, the biological activity of magnesium and its degradation products provides a plausible explanation for the regenerative outcomes observed in both preclinical and clinical studies.

Magnesium is a naturally occurring element in the human body, playing essential roles in bone metabolism and cellular function [34,48]. Magnesium membranes degrade to release magnesium ions, which stimulate periosteal and mesenchymal stem cells and osteoblasts to promote cortical bone growth, reinforcing the osteoinductive potential of magnesium-based materials beyond membranes alone [17,22]. This occurs via key signaling pathways, including the Wnt/β-catenin and JAK1-STAT3 pathways, enhancing osteogenic marker expression and matrix protein production [52,58]. In vitro evidence showed that optimal Mg^2+^ levels significantly enhance osteogenic differentiation of maxillary sinus membrane stem cells, supporting magnesium’s role in improving sinus grafting outcomes [37]. These controlled laboratory observations are consistent with a concentration-dependent biological effect and suggest a potential mechanism. However, the complex in vivo microenvironment is not replicated by in vitro conditions; the actual Mg^2+^ concentrations achieved at implant sites in human patients are unknown, and it remains unestablished whether these concentrations fall within the “optimal” range identified in cell culture.

One study reported a magnesium-reinforced collagen membrane incorporating metal–phenolic network-coated micro-magnesium wires, which modulated the osteoimmune environment by scavenging reactive oxygen species and reprogramming macrophage metabolism from glycolysis to oxidative phosphorylation via NRF2 pathway activation. This suppressed NF-κB-mediated inflammation and promoted osteogenesis in rat calvarial defect models [38].

Magnesium also promotes soft tissue adhesion, as evidenced by increased attachment of human gingival fibroblast-1 (HGF-1) cells and the formation of a continuous, well-organized cell layer on magnesium membrane surfaces, suggesting improved integration with surrounding gingival tissue during the healing process [15]. While this observation suggests potential for soft tissue integration, in vitro cell behavior on materials does not directly predict clinical tissue response in vivo, which is influenced by vascularization, wound healing phases, and systemic factors not present in culture systems.

Additionally, Magnesium membranes exhibit intrinsic antibacterial properties, largely due to the localized increase in pH and osmolarity that occurs during their degradation [47,48,52]. The antibacterial environment created during magnesium degradation suppresses bacterial colonization and biofilm formation, reducing infection risk and protecting the membrane from premature breakdown, an issue commonly observed with collagen membranes exposed to bacterial collagenase [59]. Notably, even when the magnesium membrane was left intentionally exposed, no signs of infection were observed, a finding potentially attributed to the membrane’s intrinsic antibacterial behavior, as demonstrated by Blaskovic et al. [48].

In a single clinical case, Felice et al. [54] reported that a magnesium-substituted hydroxyapatite graft achieved a mean vertical bone gain of 4.9 mm with histological integration. This one-case observation suggests that magnesium-containing materials may support bone formation.

The preclinical and in vitro evidence presented above supports the plausibility that magnesium could function as a bioactive material promoting osteogenesis. Current evidence permits only hypothesis-generating conclusions about bioactivity.

### 4.2. Degradation Behavior and the Role of the Corrosion Process

An essential factor underlying the clinical applicability of magnesium membranes is their controlled degradation behavior in physiological environments. The corrosion of magnesium in aqueous physiological media proceeds through a well-characterized electrochemical process. The overall reaction can be summarized as follows:

$$Mg+2H_{2}O\to Mg(OH{)}_{2}+H_{2}↑$$

The degradation of magnesium membranes used for guided bone regeneration is characterized by a gradual corrosion process in physiological environments, leading to the release of magnesium ions, the formation of a salty corrosion layer, and the formation of transient gas cavities [16]. The degradation kinetics of magnesium membranes follow a biphasic pattern characterized by distinct temporal phases with different corrosion rates. The initial rapid degradation phase occurs during weeks 1–8 post-implantation, driven by direct exposure of the magnesium surface to physiological fluids and the establishment of electrochemical gradients [16,28]. This is followed by a slower secondary phase continuing through week 16, during which the accumulating corrosion layer (primarily Mg(OH)_2_ and magnesium phosphates) acts as a diffusion barrier that retards further degradation [16]. Complete resorption is achieved by approximately 52 weeks [27].

The corrosion process is crucial to the membrane’s resorbability and clinical performance. As magnesium corrodes, it forms a protective layer of magnesium salts and hydroxides, which temporarily extends the membrane’s functional lifespan and space-maintaining capability [16]. The release of hydrogen gas during corrosion can result in local gas cavities, but these are typically self-limiting and do not compromise bone regeneration or tissue health [28,29]. Furthermore, hydrogen has been shown to possess antioxidant and anti-inflammatory properties through selective scavenging of reactive oxygen species, potentially modulating the local immune response and creating a favorable microenvironment for bone regeneration [55]. Preclinical evidence from Beitlitum et al. [33] provides a practical demonstration of this behavior, showing that in rabbit calvaria defects, magnesium membranes promoted bone formation while partial degradation and transient hydrogen gas formation at 8 weeks did not impair healing. However, temporary gas accumulation beneath the membrane may, in some cases, exert pressure along the suture line, potentially contributing to wound dehiscence [43]. The corrosion products are gradually resorbed and replaced by new bone, with only healthy tissue remaining after complete membrane degradation [16,56]. A study by Blašković et al. [39] addressed the potential concern regarding graft material interactions by in vitro and clinical testing, demonstrating that bovine bone graft combined with hyaluronate produced an alkaline rather than acidic environment, with no accelerated magnesium membrane degradation or adverse clinical effects, confirming compatibility between these materials.

Importantly, this degradation profile aligns with the biological timeline of guided bone regeneration, ensuring barrier function during early healing while avoiding the need for retrieval surgery.

### 4.3. Mechanical Strength, Handling, and Clinical Advantages

In vitro mechanical testing and preclinical animal studies examined whether magnesium membranes could address limitations of conventional resorbable membranes. Magnesium has extensive use in medical devices and demonstrates mechanical strength and biocompatibility in non-oral applications (cardiovascular, orthopedic) [52]. However, direct clinical comparison to collagen membranes in bone regeneration has not been performed.

Preclinical studies reported favorable in vitro mechanical properties. Rider et al. [16] conducted mechanical testing of magnesium membranes in controlled laboratory conditions, reporting a maximum tensile stress of 183 ± 10.7 megapascal (MPa). This rating exceeds published values for collagen membranes. However, (1) these comparisons are indirect (data from different studies with different testing protocols), and (2) in vitro mechanical properties do not directly predict clinical performance. Limited clinical case reports suggest that magnesium membranes may be easier to handle than collagen membranes and can be shaped, trimmed, or bent to fit defects [43,47,48,49]. These observations come from case experience rather than controlled comparative studies.

An in vivo case study demonstrated that pure magnesium membranes, combined with resorbable magnesium fixation screws, could be promising materials for GBR in humans. Their favorable mechanical properties, controlled degradability, ease of handling, and positive clinical outcomes show their potential as possible alternatives to conventional barrier membranes [27]. Additionally, experimental models show improved alveolar bone height and tissue volume with magnesium membranes, likely due to favorable mechanical support and biodegradability [36]. However, the initial postoperative period may be marked by slower mucosal healing and a heightened innate immune response, possibly related to material-associated inflammation [36].

Newer composite designs combining magnesium with polymers (3D-printed, sandwich-structured) have been developed in preclinical models to optimize mechanical stability and controlled degradation [32,37]. Similarly, Zhang et al. [22] demonstrated that incorporating a fluoride-coated AZ91 magnesium alloy core within a PLA membrane significantly enhanced mechanical properties compared to non-reinforced PLA, with the composite maintaining mechanical superiority for at least 3 weeks during degradation. The magnesium alloy core was completely degraded after 16–20 weeks, aligning with the biological timeline of guided bone regeneration. Importantly, pre-osteoblast cell viability was preserved, indicating that the accelerated degradation of the composite did not compromise cytocompatibility. This approach illustrates a broader trend toward hybrid magnesium–polymer membrane designs.

However, no clinical experience with these designs has been reported. Whether superior mechanical properties in laboratory conditions translate to clinically meaningful advantages in bone regeneration outcomes remains unknown and has not been evaluated in comparative clinical trials.

### 4.4. Surface Modifications

Surface modification strategies have been explored to further optimize the degradation kinetics and biological response of magnesium membranes.

Surface modifications, such as hydrofluoric acid treatment and biomimetic coatings, can adjust the corrosion rate, minimize gas cavity formation, and enhance cytocompatibility, enabling a controlled degradation profile that better aligns with the timing of bone healing [30,55]. Additional animal studies showed that PVD-coated magnesium membranes demonstrated acceptable biocompatibility but induced a less favorable immune profile compared with uncoated magnesium, suggesting that surface chemistry significantly influences host responses [30]. MAO-coated magnesium membranes further reduced early corrosion while maintaining osteogenic potential comparable to other barriers, highlighting the importance of coating technologies in achieving predictable clinical performance [31]. Similarly, PLA–HA–walnut shell hybrid coatings represent a sustainable, low-cost surface modification strategy that improved corrosion resistance and bioactivity of magnesium substrates in vitro, though biological cell viability testing has not yet been performed [24,25]. Alginate–CuO–MXene hybrid coatings offer a dual-function approach, combining a 77% reduction in corrosion current density with broad-spectrum antibacterial activity while preserving cytocompatibility, addressing rapid corrosion and susceptibility to infection, two major limitations of unmodified magnesium alloys [26].

These findings highlight the importance of surface engineering in tailoring magnesium membranes to achieve predictable and clinically relevant performance.

### 4.5. Performance of Magnesium Membranes and Fixation Screws in Bone Regeneration

Case reports and small case series describe clinical outcomes following magnesium membrane use. However, these represent Level 4 evidence, lacking control groups, randomization, or standardized outcome measures. Individual case reports document bone regeneration across various defect types but cannot establish efficacy, identify optimal protocols, or compare outcomes to conventional alternatives. The following examples illustrate the types of cases in which magnesium membranes have been applied but should be interpreted as observational clinical experience rather than controlled evidence.

In a clinical case by Frosecchi [41], the extraction of impacted teeth created a complex defect. The treating clinician selected a magnesium membrane for this case, and subsequent radiographic follow-up showed bone volume maintenance. Palkovics et al. [42] documented retrospectively that two patients treated with magnesium membranes achieved horizontal and vertical ridge augmentation with stable bone volume at two-year follow-up. This two-case observation is consistent with bone maintenance but does not establish efficacy, as outcomes depend on multiple factors (patient health, surgical technique, graft material, and healing capacity) beyond the membrane alone.

In a single retrospective case, one patient treated with a magnesium membrane showed stable augmented bone at one year; however, soft tissue loss around implants was observed [43].

Chaushu et al. [44] documented one case of a large cystic lesion treated with combined marsupialization, enucleation, and GBR using a magnesium membrane and xenograft. At 16-month follow-up, the patient was asymptomatic with stable teeth and preserved bone and soft tissue contours. Elad et al. [45] documented a case series of four patients in whom magnesium membranes were applied to repair perforated Schneiderian membranes and support bone grafting using combined xenograft and allograft materials. At follow-up, newly formed alveolar bone was observed, with radiographic vertical bone gains ranging from approximately 10 mm to 15–20 mm. Complete membrane resorption was confirmed, and both vertical and horizontal bone augmentations appeared stable on imaging. Implant placement was subsequently carried out at all treated sites. However, this four-case observation documented bone formation specifically in sinus lift procedures but does not establish whether magnesium membranes are superior to conventional materials for this indication or identify which factors, such as magnesium properties, graft material, surgical technique, or patient healing capacity, contributed to the outcomes. No control group or comparison to conventional membranes was included, so whether magnesium membranes provide an advantage over alternative materials cannot be determined in these cases. Hangyasi et al. [47] documented a case series of three patients with intrabony periodontal defects treated with customized magnesium membranes shaped into different forms (strips, T-shapes, and M-shapes) to match defect morphology. Following 4–6 months of healing, radiographic analysis showed bone fill within the defect, and clinical probing measurements indicated an average reduction in periodontal probing depth of 1.66 ± 0.29 mm compared to baseline. Soft-tissue healing was noted to be favorable, and no major complications were documented during the follow-up period. The improvement in probing depth could result from multiple mechanisms: magnesium membrane properties, new bone formation, connective tissue reattachment, changes in gingival inflammation, improved patient oral hygiene, or combinations thereof. Beyond conventional GBR indications, a structured minimally invasive approach termed the Magnesium Membrane Shield Technique has been described for the management of severe buccal bone deficiency in the aesthetic zone [51]. This technique leverages the mechanical stability of the magnesium membrane to maintain buccal contour while gradually resorbing, avoiding secondary surgery and its associated morbidity. The reported case demonstrated uneventful healing with satisfactory functional and aesthetic outcomes. Buccal bone preservation is particularly critical in the aesthetic zone, where bone volume directly influences soft tissue contour and long-term implant esthetics.

Overall, the available clinical evidence suggests that magnesium membranes exhibit a favorable safety profile with predominantly minor and self-limiting complications.

Through the included studies, magnesium membranes consistently demonstrated a favorable clinical safety profile, with most cases showing uneventful healing and no signs of infection or graft failure [41,42,44,45,47,48,49]. Soft-tissue healing was generally stable, and the membranes provided sufficient mechanical support to maintain regenerative space during early healing, supporting maintenance of regenerative space during healing across different defect types [42,44,45].

A central topic in the literature concerns soft-tissue complications, particularly membrane exposure. Among all studies, Tabanella et al. [50] documented the most significant soft-tissue issues, reporting varying severity of wound dehiscences in all four cases. These ranged from minor exposures less than 3 mm to extensive exposures that revealed up to three-quarters of the membrane. Despite the magnitude of these complications, no infections occurred, and both vertical and horizontal bone gains were confirmed at re-entry, demonstrating that magnesium membranes can maintain their regenerative role even under open-healing conditions [50]. However, localized necrosis and discomfort caused by a loose screw were noted, indicating that although magnesium membranes tolerate exposure better than collagen, soft-tissue handling remains clinically important [50,56].

In contrast, Franke et al. [43] reported only minor, self-limiting complications during membrane degradation. The patient described a brief “prickly” sensation during the first two postoperative weeks, likely related to hydrogen gas release as the membrane and screws resorbed. This effect required no intervention and resolved spontaneously. At three weeks, partial screw visibility was observed, but complete soft-tissue healing occurred by three months with a stable zone of keratinized mucosa [43]. At the one-year follow-up, bone volume remained preserved, confirming that early transient symptoms did not compromise the regenerative outcome [43].

Taken together, these observations suggest that the effectiveness of magnesium membranes is not limited to specific clinical scenarios but reflects a broadly applicable combination of mechanical stability and controlled resorption. However, these case reports documented what occurred but do not establish that magnesium membranes were the primary determinant of outcomes. Thus, while case reports provide preliminary observational experience, they are insufficient to establish clinical efficacy, standardized protocols, or superiority over conventional materials.

### 4.6. Comparative Considerations with Conventional Barrier Materials

The clinical relevance of magnesium membranes should be contextualized against established alternatives. Resorbable collagen membranes offer biocompatibility but lack mechanical rigidity for vertical augmentation and are susceptible to premature enzymatic degradation by bacterial collagenase when exposed to the oral environment [16,28]. Non-resorbable titanium meshes provide excellent space maintenance but necessitate a second retrieval surgery, increasing morbidity, cost, and risk of bone loss during removal [28,57]. Magnesium membranes may occupy a middle ground, offering mechanical strength superior to collagen (183 ± 10.7 MPa in preclinical testing), intrinsic antibacterial properties, and late resorption, eliminating the need for removal surgery [16,55].

However, these comparisons remain indirect, as no randomized controlled trials or prospective comparative studies have directly evaluated magnesium membranes against conventional membranes such as collagen or titanium mesh in bone regeneration outcomes.

### 4.7. Study Limitations

This scoping review has several important limitations that should be carefully considered when interpreting the findings. First, the majority of included studies represent low-level evidence (CEBM Levels 4–5), consisting predominantly of case reports, small case series, and preclinical in vivo or in vitro investigations. Additionally, the Oxford CEBM hierarchy was designed for clinical research questions, and its application to preclinical and in vitro studies represents a methodological limitation, as controlled preclinical experiments and expert opinion are classified identically as Level 5 despite representing epistemologically distinct evidence types. This reflects the early translational stage of magnesium membrane technology in oral and maxillofacial regeneration but substantially limits the strength of clinical inference. The absence of randomized controlled trials or well-designed comparative clinical studies prevents robust comparison with established barrier membranes, such as collagen or titanium-reinforced systems, and precludes definitive conclusions regarding relative efficacy, safety, and long-term outcomes.

Second, there was substantial heterogeneity across the included studies with respect to defect type, surgical indication, membrane design, fixation method, grafting materials, and follow-up duration. Outcome measures were inconsistently reported and ranged from qualitative clinical observations to radiographic assessments, histology, or histomorphometry, limiting cross-study comparability and synthesis. In addition, sample sizes were generally small, particularly in clinical reports, increasing the risk of selection bias and limiting generalizability.

Third, many preclinical studies employed short observation periods, which may not fully capture long-term degradation behavior, late tissue responses, or potential delayed complications associated with magnesium corrosion and hydrogen gas release.

Finally, as a scoping review, this study did not include a formal risk-of-bias assessment or quantitative synthesis, which limits the ability to evaluate methodological quality or effect size. While the overall findings consistently support the biological plausibility and regenerative potential of magnesium membranes, the current body of evidence should be considered hypothesis-generating rather than practice-defining, underscoring the need for larger, well-controlled clinical trials with standardized protocols, longer follow-up periods, and clearly defined outcome measures.

## 5. Conclusions

Magnesium membranes have been investigated as a potential material for guided bone regeneration. Currently available evidence, consisting of 30 studies with 63% Level 5 (preclinical and in vitro studies) and 37% Level 4 (case reports and case series) evidence, documents the occurrence of bone regeneration in case reports and demonstrates in vitro and animal model evidence for plausible biological mechanisms. Case reports describe favorable handling and bone outcomes. However, without control groups, randomization, or systematic adverse event monitoring, these observations do not establish clinical efficacy or superiority over conventional membranes. Reported complications in case series were generally minor. Nevertheless, a comprehensive safety assessment requires a prospective comparative study.

Preclinical evidence suggests potential biological activity through magnesium ion release and pH modulation. These mechanistic findings are hypothesis-generating and warrant further investigation but cannot yet be translated to defined clinical protocols or guaranteed outcomes in human patients.

In conclusion, magnesium membranes represent an area of active early-stage investigation with sufficient preliminary evidence to justify prospective clinical trials. However, current evidence is insufficient to establish clinical efficacy, recommend magnesium over conventional materials, or define standardized clinical protocols. Further high-quality prospective, randomized, controlled clinical trials with standardized outcome measures and adequate follow-up are essential before magnesium membranes can be considered an evidence-based advancement in guided bone regeneration.

## Figures and Tables

**Table 1:** Electronic databases and search terms.

Electronic Database	Search Term
PubMed	“Bone Regeneration” OR “Alveolar Bone Grafting” OR “Tissue Regeneration” AND “Magnesium”. (“Bone Regeneration” OR “Alveolar Bone Grafting” OR “Tissue Regeneration” OR “Guided Bone Regeneration” OR “GBR” OR “Ridge Augmentation” OR “Alveolar Ridge Augmentation”) AND (“Magnesium” OR “Mg” OR “Magnesium alloy” OR “Magnesium-based”) AND (“Membrane” OR “Barrier Membrane” OR “Resorbable Membrane” OR “Biodegradable Membrane” OR “Barrier” OR “Mesh”).
ScienceDirect	“Bone Regeneration” OR “Alveolar Bone Grafting” OR “Tissue Regeneration” AND “Magnesium”. (“Bone Regeneration” OR “Alveolar Bone Grafting” OR “Tissue Regeneration” OR “Guided Bone Regeneration” OR “GBR” OR “Ridge Augmentation” OR “Alveolar Ridge Augmentation”) AND (“Magnesium” OR “Mg” OR “Magnesium alloy” OR “Magnesium-based”) AND (“Membrane” OR “Barrier Membrane” OR “Resorbable Membrane” OR “Biodegradable Membrane” OR “Barrier” OR “Mesh”).
Scopus	“Bone Regeneration” OR “Alveolar Bone Grafting” OR “Tissue Regeneration” AND “Magnesium”. (“Bone Regeneration” OR “Alveolar Bone Grafting” OR “Tissue Regeneration” OR “Guided Bone Regeneration” OR “GBR” OR “Ridge Augmentation” OR “Alveolar Ridge Augmentation”) AND (“Magnesium” OR “Mg” OR “Magnesium alloy” OR “Magnesium-based”) AND (“Membrane” OR “Barrier Membrane” OR “Resorbable Membrane” OR “Biodegradable Membrane” OR “Barrier” OR “Mesh”).
Web of Science	“Bone Regeneration” OR “Alveolar Bone Grafting” OR “Tissue Regeneration” AND “Magnesium”. (“Bone Regeneration” OR “Alveolar Bone Grafting” OR “Tissue Regeneration” OR “Guided Bone Regeneration” OR “GBR” OR “Ridge Augmentation” OR “Alveolar Ridge Augmentation”) AND (“Magnesium” OR “Mg” OR “Magnesium alloy” OR “Magnesium-based”) AND (“Membrane” OR “Barrier Membrane” OR “Resorbable Membrane” OR “Biodegradable Membrane” OR “Barrier” OR “Mesh”).
Cochrane	“Bone Regeneration” OR “Alveolar Bone Grafting” OR “Tissue Regeneration” AND “Magnesium”. (“Bone Regeneration” OR “Alveolar Bone Grafting” OR “Tissue Regeneration” OR “Guided Bone Regeneration” OR “GBR” OR “Ridge Augmentation” OR “Alveolar Ridge Augmentation”) AND (“Magnesium” OR “Mg” OR “Magnesium alloy” OR “Magnesium-based”) AND (“Membrane” OR “Barrier Membrane” OR “Resorbable Membrane” OR “Biodegradable Membrane” OR “Barrier” OR “Mesh”).
Google Scholar	“Bone Regeneration” OR “Alveolar Bone Grafting” OR “Tissue Regeneration” AND “Magnesium”. (“Bone Regeneration” OR “Alveolar Bone Grafting” OR “Tissue Regeneration” OR “Guided Bone Regeneration” OR “GBR” OR “Ridge Augmentation” OR “Alveolar Ridge Augmentation”) AND (“Magnesium” OR “Mg” OR “Magnesium alloy” OR “Magnesium-based”) AND (“Membrane” OR “Barrier Membrane” OR “Resorbable Membrane” OR “Biodegradable Membrane” OR “Barrier” OR “Mesh”).

**Table 2:** Characteristics of included studies.

Author	Title	Type of Study	CEBM Level	Sample Size	Main Outcome
Chen et al. (2022) [14]	Recent Advances in the Development of Magnesium-Based Alloy Guided Bone Regeneration (GBR) Membrane	Narrative review	5		Magnesium-based membranes offer strength, biodegradability, and osteogenic and antibacterial effects; challenges like rapid corrosion, hydrogen release, and stress corrosion are addressed via alloying and surface modifications.
Rider et al. (2021) [15]	Biodegradable magnesium barrier membrane used for guided bone regeneration in dental surgery	Original research in vivo	4	20	The pure magnesium membrane-maintained barrier function and space during early healing showed controlled degradation and achieved bone regeneration comparable to collagen membranes without adverse reactions.
Amberg et al. (2018) [17]	Design of a migration assay for human gingival fibroblasts on biodegradable magnesium surfaces	In vitro study	5		A reproducible assay demonstrated that human gingival fibroblasts attach to and migrate on biodegradable magnesium substrates.
Zhang et al. (2025) [22]	Impact of Strontium, Magnesium, and Zinc Ions on the In Vitro Osteogenesis of Maxillary Sinus Membrane Stem Cells	in vitro study	5		Moderate Mg^2+^ concentrations increased expression of osteogenic differentiation markers in vitro.
Malaiappan et al. (2025) [23]	Osteogenic Potential of Magnesium Oxide Nanoparticles in Bone Regeneration: A Systematic Review	Systematic review	5		Reported that MgO nanoparticles might support osteoblast activity and osteogenic gene expression in vitro and promote bone formation, density, and implant integration in vivo, supporting bone regeneration.
Zhang et al. (2020) [24]	Bioresorbable magnesium-reinforced PLA membrane for guided bone/tissue regeneration	Preclinical in vivo and in vitro research	5		Improved mechanical strength, controlled degradation, and maintained cytocompatibility
Topuz et al. (2025) [25]	Sustainable walnut shell-filled PLA–HA coatings for Mg biomaterials	in vitro study		5	Improved corrosion resistance and bioactivity of Mg biomaterials via hybrid polymer coating
Karataş et al. (2026) [26]	MXene and CuO modified alginate coatings for AZ31 Mg alloy	in vitro study	5		Enhanced corrosion resistance, antibacterial activity, and cytocompatibility of Mg alloy
Rider et al. (2022) [27]	Biodegradation of a Magnesium Alloy Fixation Screw Used in a Guided Bone Regeneration Model in Beagle Dogs	Original research in vivo	5	20	Magnesium screws provided bone regeneration comparable to titanium, with gradual resorption and only transient, non-interfering tissue reactions.
Rider et al. (2022) [28]	Analysis of a Pure Magnesium Membrane Degradation Process and Its Functionality When Used in a Guided Bone Regeneration Model in Beagle Dogs	Original research in vivo	5	18	Micro-CT * showed bone formation comparable to collagen membranes, with controlled magnesium degradation and no long-term adverse tissue reactions.
Barbeck et al. (2020) [29]	Tissue Response of an Innovative Volume Stable Magnesium-Supported GBR/GTR Barrier Membrane	Original research in vivo	5		The magnesium-supported membrane showed controlled degradation, favorable tissue response, maintained early volume stability, and was associated with bone formation in preclinical model.
Steigmann et al. (2020) [30]	Biocompatibility and Immune Response of a Newly Developed Volume-Stable Magnesium-Based Barrier Membrane in Combination with a PVD Coating for Guided Bone Regeneration (GBR)	Preclinical in vivo and in vitro research	5		Despite poor in vitro cytocompatibility, both membranes showed acceptable in vivo biocompatibility; uncoated magnesium elicited a collagen-like immune response, while PVD ** coating showed no added benefit.
Shan et al. (2022) [31]	Degradable Pure Magnesium Used as a Barrier Film for Oral Bone Regeneration	preclinical in vivo and in vitro research	5		MAO ^#^-coated magnesium showed controlled degradation, supported osteoblast activity, and achieved bone regeneration comparable to titanium membranes.
Wang et al. (2025) [32]	Magnesium-reinforced sandwich-structured composite membranes promote osteogenesis	Preclinical in vivo and in vitro research	5		Magnesium-reinforced sandwich-structured composite membranes provided improved mechanical stability and reported osteoblast activity and osteogenic gene expression through sustained Mg^2+^ release, supporting bone regeneration in preclinical models.
Beitilitum et al. (2025) [33]	Magnesium Resorbable Membrane for Guided Bone Regeneration in Critical Size Defect Model in Rabbits—Histomorphometric Analysis	Original research in vivo	5	9	Mg membrane was associated with greater bone formation in rabbit calvarial critical-size defects, with greater histomorphometric new bone compared to non-membrane controls, despite transient gas accumulation due to degradation.
Vujović et al. (2022) [34]	Applications of Biodegradable Magnesium-Based Materials in Reconstructive Oral and Maxillofacial Surgery: A Review.	Narrative review	5		Mg-based biomaterials show promising biodegradability, mechanical strength, and osteogenic potential in oral and maxillofacial reconstruction, but clinical evidence remains limited.
Yan et al. (2022) [35]	Feasibility and efficacy of a degradable magnesium-alloy GBR membrane for bone augmentation in a distal bone-defect model in beagle dogs	Original research in vivo	5		Suggested feasibility and was associated with bone formation in an animal model
Wu et al. (2025) [36]	Pure Magnesium GBR Membrane Affects Oral Tissue Regeneration.	Original research in vivo	5		Pure Mg membranes yielded greater bone formation in a preclinical model.
Liu et al. (2024) [37]	A 3D printed magnesium ammonium phosphate/polycaprolactone composite membrane for Guided bone regeneration	Preclinical in vivo and in vitro research	5		A 3D-printed magnesium ammonium phosphate/polycaprolactone composite membrane demonstrated acceptable mechanical properties, controlled degradation, and sustained magnesium ion release and showed osteogenic differentiation and bone formation in preclinical models.
Mu et al. (2026) [38]	Osteoimmunometabolic modulation via hydrogen-self-supplying magnesium-reinforced collagen membrane for enhanced GBR	Original research in vivo	5		Demonstrated bone regeneration via immunomodulation, ROS scavenging, and macrophage metabolic reprogramming
Blašković et al. (2026) [39]	Evaluation between biodegradable magnesium metal GBR membrane and bovine graft with or without hyaluronate	Case series	4	2	Bone regeneration was observed, demonstrating compatibility of the Mg membrane with graft materials and hyaluronate
Elad et al. (2023) [40]	Application of Biodegradable Magnesium Membrane Shield Technique for Immediate Dentoalveolar Bone Regeneration	Case series	4	4	All cases demonstrated bone regeneration and soft-tissue healing, with follow-up imaging showing thick cortical bone formation in some sites. The membrane was adaptable to defect shape and could be applied as a single or double layer for mechanical support. Its biodegradable nature eliminated the need for secondary removal surgery.
Frosecchi et al. (2023) [41]	Horizontal and Vertical Defect Management with a Novel Degradable Pure Magnesium Guided Bone Regeneration (GBR) Membrane—A Clinical Case	Clinical case	4	1	Complex defect was augmented with bovine graft and magnesium ‘arch’ membrane; over 8 months, volume was maintained, healing was complication-free, membrane resorbed, and implants successfully placed
Palkovics et al. (2023) [42]	Possible Applications for a Biodegradable Magnesium Membrane in Alveolar Ridge Augmentation–Retrospective Case Report with Two Years of Follow-Up	Retrospective case report	4	2	Magnesium membranes achieved 0.12–0.36 cm^3^ bone gain with horizontal and vertical improvements; two-year follow-up showed stable volume, no peri-implant bone loss, and effective space maintenance in challenging defects.
Franke et al. (2024) [43]	Guided Bone Regeneration in the Posterior Mandible Using a Resorbable Metal Magnesium Membrane and Fixation Screws: A Case Report	Clinical case	5	1	The membrane supported bone graft consolidation, and at 3 months, sufficient bone volume and quality were observed for implant placement. The membrane fully resorbed, and the augmented site healed without complications, despite minor transient soft-tissue effects.
Chaushu et al. (2025) [44]	Use of a Resorbable Magnesium Membrane for Bone Regeneration After Large Radicular Cyst Removal: A Clinical Case Report	Clinical case	4	1	At 16 months, CBCT ^++^ showed restored palatal bone and cortical formation; teeth were asymptomatic with healthy tissues. The resorbable magnesium membrane supported graft stability and avoided removal surgery.
Elad et al. (2023) [45]	Resorbable magnesium metal membrane for sinus lift procedures: a case series	Case series	4	4	In all four cases, the magnesium membrane was used to repair or replace the sinus membrane and support bone grafts in the sinus cavity. Healing resulted in newly formed alveolar bone with height gains of 10-20 mm, and the magnesium membrane was fully resorbed. Vertical and horizontal bone augmentation remained stable, providing sufficient regenerated bone to support dental implants.
Lv et al. (2025) [46]	Bolstered bone regeneration by multiscale customized magnesium scaffolds with hierarchical structures and tempered degradation	Preclinical in vivo and in vitro research	5		The magnesium scaffold provided cancellous bone-like strength, provided osteoconductive support, and increased in vivo bone formation.
Hangyasi et al. (2023) [47]	Regeneration of Intrabony Defects Using a Novel Magnesium Membrane	Case series	4	3	In all three cases, the magnesium membrane could be easily shaped into customized forms (strip, T-shape, M-shape) to adapt to the specific morphology of each intrabony defect. After 4–6 months of healing, radiological analysis identified bone fill within the treated defects and periodontal probing depth reduction by an average of 1.66 ± 0.29 mm, indicating a gain in bone support. Soft-tissue healing was favorable, and no major complications were reported during the healing period.
Blaskovic et al. (2024) [48]	Magnesium Membrane Shield Technique for Alveolar Ridge Preservation: Step-by-Step Representative Case Report of Buccal Bone Wall Dehiscence with Clinical and Histological Evaluations	Clinical case	4	1	After six months, sufficient bone volume allowed implant placement. Histology showed ~47% new bone, ~19% residual graft, and no inflammation, with active remodeling at the bone-biomaterial interface. Soft tissue healed well, and the final restoration achieved good esthetic and functional outcomes.
Blaskovic et al. (2023) [49]	Guided Bone Regeneration Using a Novel Magnesium Membrane: A Literature Review and a Report of Two Cases in Humans	Literature review and clinical cases	4	2	Magnesium membranes provide strength, biocompatibility, degradability, and barrier function; human cases showed stable grafts, complication-free healing, and complete membrane resorption with satisfactory bone regeneration.
Tabanella et al. (2025) [50]	Open Wound Healing in Guided Bone Regeneration Using a Magnesium Membrane: A Paradigm Shift	Case series	4	4	Despite membrane exposure in all cases, from small to large, none of the patients experienced pain, infection, or other clinical complications. Implant placement was carried out as planned, and importantly, there was no significant bone loss observed despite exposure. The resorbable magnesium membrane maintained its barrier function and preserved the augmented bone volume, even under open-wound healing conditions.
Tabanella et al. (2026) [51]	Magnesium membrane shield technique for buccal bone deficiency	Clinical case	4	1	Successful buccal bone regeneration with favorable aesthetic and functional outcomes
Witte et al. (2010) [52]	The history of biodegradable magnesium implants: a review	Narrative review	5		Magnesium-based implants show biodegradability and biocompatibility; challenges like corrosion and mechanical strength are partly addressed by alloying and surface treatments.
Li et al. (2025) [53]	Magnesium-based barrier membrane for guided bone regeneration: From bedside to bench and back again	Narrative review	5		Mg-based membranes support GBR ^+^ principles, promote bone regeneration, prevent bacterial infiltration, enhance clinical safety, and extend use beyond traditional GBR, even in wound complications
Felice et al. (2013) [54]	Magnesium-substituted hydroxyapatite grafting using the vertical inlay technique	Clinical case	4	1	After three months, vertical bone gain of 4.9 mm was achieved at implant placement. Histology showed that the grafted Mg-HA material was fully infiltrated by new bone, demonstrating integration into living tissue. Implants were restored with provisional and definitive prostheses at four and eight months, respectively, without complications.
Wu et al. (2019) [55]	Surface modification of pure magnesium mesh for GBR	Original research in vivo	5		Surface-modified Mg mesh was associated with bone formation and acceptable biocompatibility in a preclinical model
Khalil et al. (2025) [56]	Surface Treatment With Cell Culture Medium: A Biomimetic Approach to Enhance the Resistance to Biocorrosion in Mg and Mg-Based Alloys—A Review	Narrative review	5		DMEM ^##^ promotes calcium phosphate layer formation on Mg implants, enhancing corrosion resistance and bone-like environments; synthetic buffers accelerate corrosion, and protein media risk contamination. Layer stability depends on fluid dynamics, while long-term mechanical and in vivo effects remain unclear.
Lacin et al. (2026) [57]	Magnesium-based resorbable biomaterials: Biological effects to clinical use	Narrative review		5	Summarizes biological effects, degradation behavior, and clinical potential of Mg-based biomaterials

* CT—computed tomography. ** PVD—physical vapor deposition. ^#^ MAO—micro arc oxidation. ^##^ DMEM—Dulbecco’s Modified Eagle Medium. ^+^ GBR—guided bone regeneration. ^++^ CBCT—cone-beam computed tomography.

## Data Availability

No new data were created or analyzed in this study. Data sharing is not applicable to this article.

## Supplementary Materials

The following supporting information can be downloaded at: https://www.mdpi.com/article/10.3390/jfb17060293/s1, Table S1: Preferred Reporting Items for Systematic reviews and Meta-Analyses extension for Scoping Reviews (PRISMA-ScR) Checklist.

## Abbreviations

The following abbreviations are used in this manuscript:

GBR	Guided Bone Regeneration
e-PTFE	Expanded Polytetrafluoroethylene
HGF-1	Human Gingival Fibroblast 1
Mpa	Megapascal
Mg	Magnesium
CT	Computed Tomography
PVD	Physical Vapor Deposition
MAO	Micro Arc Oxidation
DMEM	Dulbecco’s Modified Eagle Medium
CBCT	Cone-Beam Computed Tomography
